# Tailoring Ag–Pt nanoalloys through solid-state dewetting: structural and optical insights

**DOI:** 10.3762/bjnano.17.52

**Published:** 2026-06-10

**Authors:** Marcin Łapiński, Piotr Okoczuk, Blaž Grobiša, Ewa Pawlikowska, Amelia Rozwadowska, Wojciech Sadowski, Barbara Kościelska

**Affiliations:** 1 Institute of Nanotechnology and Materials Engineering, Advanced Materials Center, Gdansk University of Technology, ul. G. Narutowicza 11/12, 80-233 Gdańsk, Poland

**Keywords:** Ag–Pt alloy, dewetting, nanoalloys, plasmon resonance, thin films

## Abstract

In this work, a cost-effective method for fabricating silver–platinum nanoalloys through thermally activated dewetting of thin metallic films is demonstrated. Ag and Pt bilayers with a total thickness of 8 nm were deposited using DC magnetron sputtering, followed by annealing at 650 °C under an argon atmosphere. The process induced the transformation of continuous bilayers into isolated nanoislands through solid-state dewetting. Scanning electron microscopy and transmission electron microscopy analyses revealed the formation of well-defined, nearly spherical nanoislands with a homogeneous elemental distribution, as measured by energy dispersive spectroscopy. Additionally, X-ray photoelectron spectroscopy measurements confirmed the coexistence of both metals in metallic states, with a slight Ag deficiency attributed to its higher instability and desorption during the annealing process. Optical measurements revealed the presence of a single resonance peak. The composition-dependent plasmonic resonance band was observed for low Pt contents, while progressively blue-shifting and decreasing in intensity with increasing Pt concentration. This behavior is consistent with the strong d-electron contribution of platinum, which suppresses plasmonic oscillations. The obtained results demonstrate that thermally activated dewetting enables the synthesis of homogeneous Ag–Pt nanoalloys at the nanoscale, both in volume and on the surface of nanostructures, overcoming miscibility limitations of the bulk Ag–Pt system, and provide insight into their structure–property relationships relevant for catalytic and plasmonic applications.

## Introduction

Nanoalloys in the form of nanostructures exhibit novel and intriguing physical and chemical properties. This phenomenon arises due to their exceptionally high surface area relative to the number of atoms in their volume, as well as the influence of quantum effects [1–2]. Notably, nanoalloys facilitate the formation of unique phases and systems that are often unattainable in macroscopic alloy systems (e.g., Ag–Pt nanoalloys [3–4] or Ni–Au [5–6]). Furthermore, they enable the formation of various crystalline arrangements and structures.

There are various methods for synthesizing metallic nanoalloys, both on solid substrates and in solution [7–9]. An auspicious method seems to be thermally activated dewetting of thin metal films deposited on glass or other substrates [9–12]. This method is noteworthy due to its cost-effectiveness, making it a compelling alternative for various applications. From a thermodynamic perspective, continuous metallic layers with nanoscale thickness can be metastable or even intrinsically unstable. This instability leads to the gradual disintegration of the continuous film, and, consequently, the formation of isolated nanoscale islands [13–16]. This process occurs at temperatures lower than the melting point of the thin film. The disintegration mechanism is driven by atomic diffusion, and the tendency of the system is to reduce the surface energy through the formation of isolated islands [17–20]. This process is governed by the pursuit of balance between the energy needed to create a new surface (surface tension) and the energy gained by reducing the total contact area between the film and the substrate (interfacial tension).

A comprehensive analysis of the transformation of a thin layer into isolated islands was provided in a series of publications by Thompson and collaborators [13,20–22]. They postulated a mechanism based on the dewetting phenomenon. According to their model, the disintegration of the film initiates at pre-existing discontinuities or voids, which can be associated with nanoscale irregularities at intergranular boundaries. A key factor governing the disintegration process of thin metallic layers is their thickness: the thinner the layer, the lower the temperature at which it transforms into isolated islands. It can be stated that the significant influence of surface energy in thin-film structures, phase separation boundaries (layer–substrate, layer–liquid phase), grain boundaries in polycrystalline structures, various defects, mainly linear and plane (e.g., twinning), or finally local inhomogeneities of the thickness of thin films, strongly modify the phase equilibrium system. This, in turn, affects critical properties, including the melting temperature of metallic structures, an effect that becomes particularly pronounced at the nanoscale. Consequently, these factors create opportunities for thermal-kinetic control, enabling the manipulation of thin-film continuity and facilitating their disintegration.

Our earlier studies have shown that thermally activated disintegration can be used to fabricate single-metal nanostructures, nanoalloys, and nanocomposites on various substrates [23–26]. In this paper, we present a method for manufacturing Ag–Pt nanoalloys by thermal disintegration of thin metallic films. This nanoalloy system is particularly significant due to its high gas-absorption capacity, making it highly relevant for catalytic applications. According to phase diagrams, bulk Ag–Pt bimetallic systems exhibit a large miscibility gap at temperatures below approximately 1190 °C, allowing alloy formation only at very high atomic concentrations of either Ag or Pt [27–29]. However, recent studies have reported the formation of Ag–Pt nanoalloys with nearly equal ratios of Ag and Pt atoms, supported by both theoretical calculations [30–32] and experimental investigations [27,33–35], raising several important questions worth answering. Observations of alloy formation at the nanoscale confirm that the miscibility between different metals can significantly increase as particle size decreases [3–6,33]. Nevertheless, in the case of nanoalloy formation induced by thermal dewetting of alternately deposited thin metallic layers, it is essential to determine whether the atoms forming the nanoalloy mix to the same extent at the surface as within the entire volume of an individual nanostructure [25–26].

Whether a nanostructure forms a genuine atomic-scale nanoalloy or a nanocomposite has a major impact on its plasmonic and catalytic properties, and thus, the application potential of the fabricated nanosystem. Moreover, when designing plasmonic devices based on Ag–Pt nanoalloys, one must take into account the damping of the plasmon resonance in platinum alloys. This phenomenon was theoretically investigated [36–39] and supported by a few experimental studies [40–41]. However, in this work, we address the question of the threshold Pt content at which its influence on the resonance position remains observable. Upon addition of Pt to the Ag nanostructures, intense d-electron excitations of Pt increase the dielectric function components, causing quenching of the plasmon resonance. We demonstrate that the Ag plasmon resonance is damped once the compositional threshold is reached.

Taking into consideration both the intrinsic challenges of Ag–Pt nanoalloy formation and their promising application potential, developing an optimized and cost-effective fabrication method remains a critical objective. In this work, we present a detailed analysis of the structure of the obtained nanoalloys, both within the volume of the nanostructures and at their surfaces, as well as the influence of Pt content in Ag–Pt nanoalloys on their plasmonic properties.

## Results and Discussion

**Figure 1:**
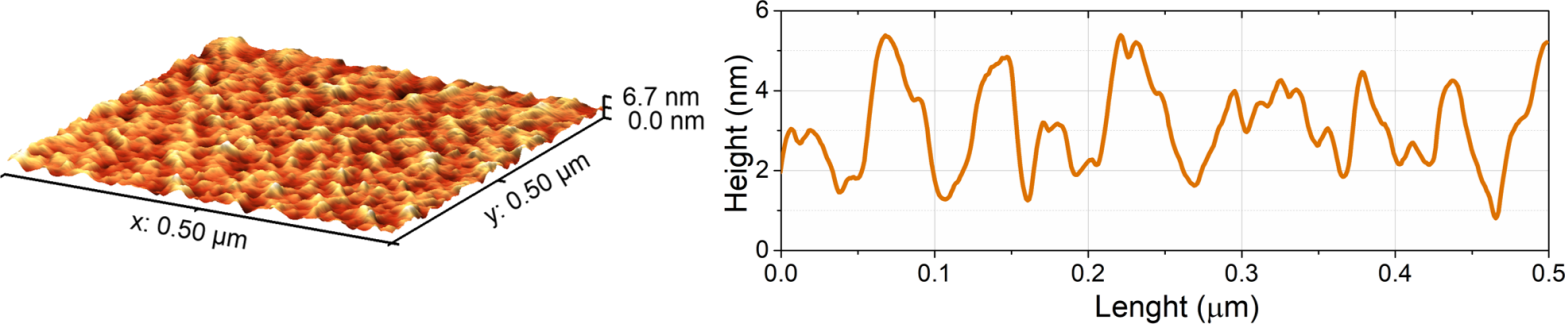
AFM image and profile of the as-deposited 4 nm Ag and 4 nm Pt bilayer.

**Figure 2:**
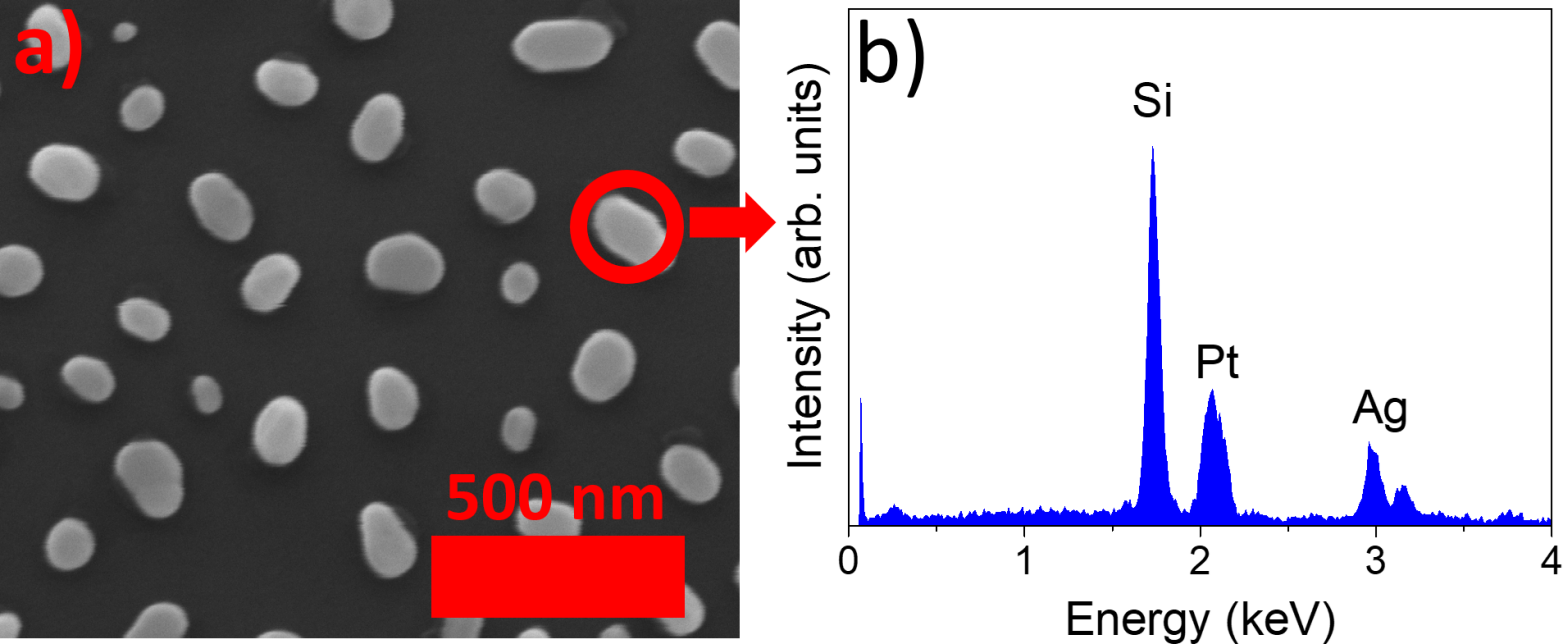
a) SEM image of nanostructures obtained by annealing at 650 °C for 15 min. 4 nm Ag and 4 nm Pt bilayer; b) results of the EDS analysis in a selected region.

As-deposited thin Ag–Pt bilayers exhibit a granular morphology, as evidenced by atomic force microscopy (AFM) measurements shown in Figure 1. This granular character leads to instability of the layers at higher temperatures, causing cracking of the continuous film in the multiple grain junctions and transformation into isolated islands. These phenomena are consistent with our earlier observations [23–24] and those of others [42–44]. It is worth noting that the nucleation of holes can also be accelerated by surface melting or premelting, especially in a region with a lower film thickness at grain boundaries, which is well visible on the linear AFM profile in Figure 1 [45–47]. Figure 2a presents an exemplary scanning electron microscopy (SEM) image of nanostructures formed as a result of annealing of a silver–platinum bilayer with a thickness of 4 nm each at 650 °C for 15 min. These annealing parameters correspond to the lowest temperature at which well-defined, nearly spherical, isolated islands were formed. These findings demonstrate that the applied annealing temperature and duration provide sufficient thermal energy to drive the decomposition of the thin Ag/Pt bilayer into isolated homogenous nanoislands. The chemical composition of the nanostructures was examined using energy dispersive spectroscopy (EDS) in point analysis mode. The example spectrum, shown in Figure 2b, confirms the coexistence of Ag and Pt in the analyzed nanostructure. The estimated compositional ratio of Ag and Pt was approximately 48 atom % Ag and 52 atom % Pt, which perfectly matches the theoretical prediction presented below in Figure 10b. Measurements for other compositions are also consistent with the theoretical concentrations of Ag and Pt. The results of the EDS measurements are presented below in Figure 10b and marked by blue triangles.

**Figure 3:**
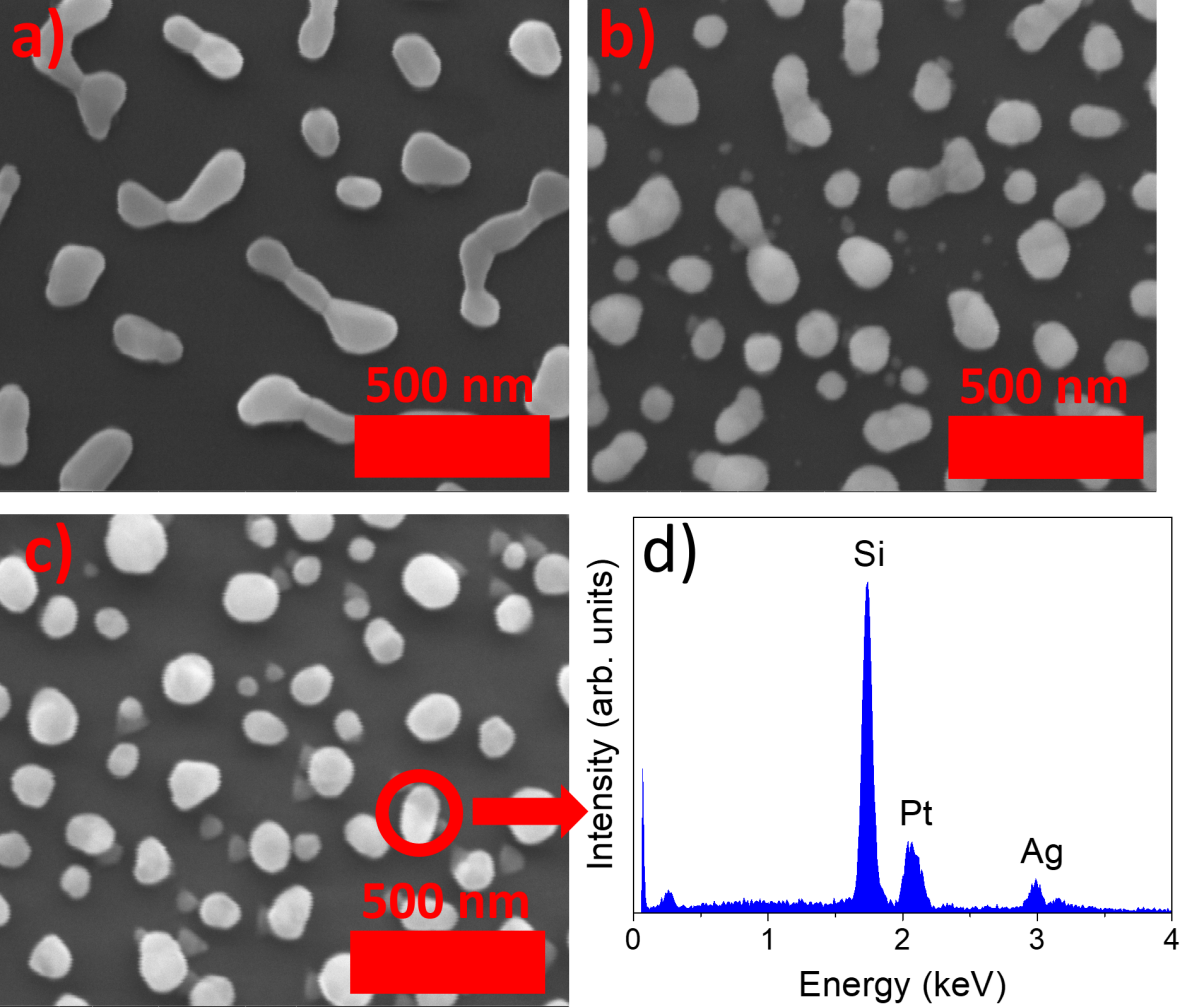
SEM images of nanostructures obtained by annealing of 5 nm Ag and 5 nm Pt bilayer for 15 min. at: a) 650 °C, b) 675 °C, c) 700 °C. d) Results of the EDS analysis in a selected region of the nanostructures obtained by annealing of the 10 nm Ag–Pt bilayer at 700 °C.

**Figure 4:**
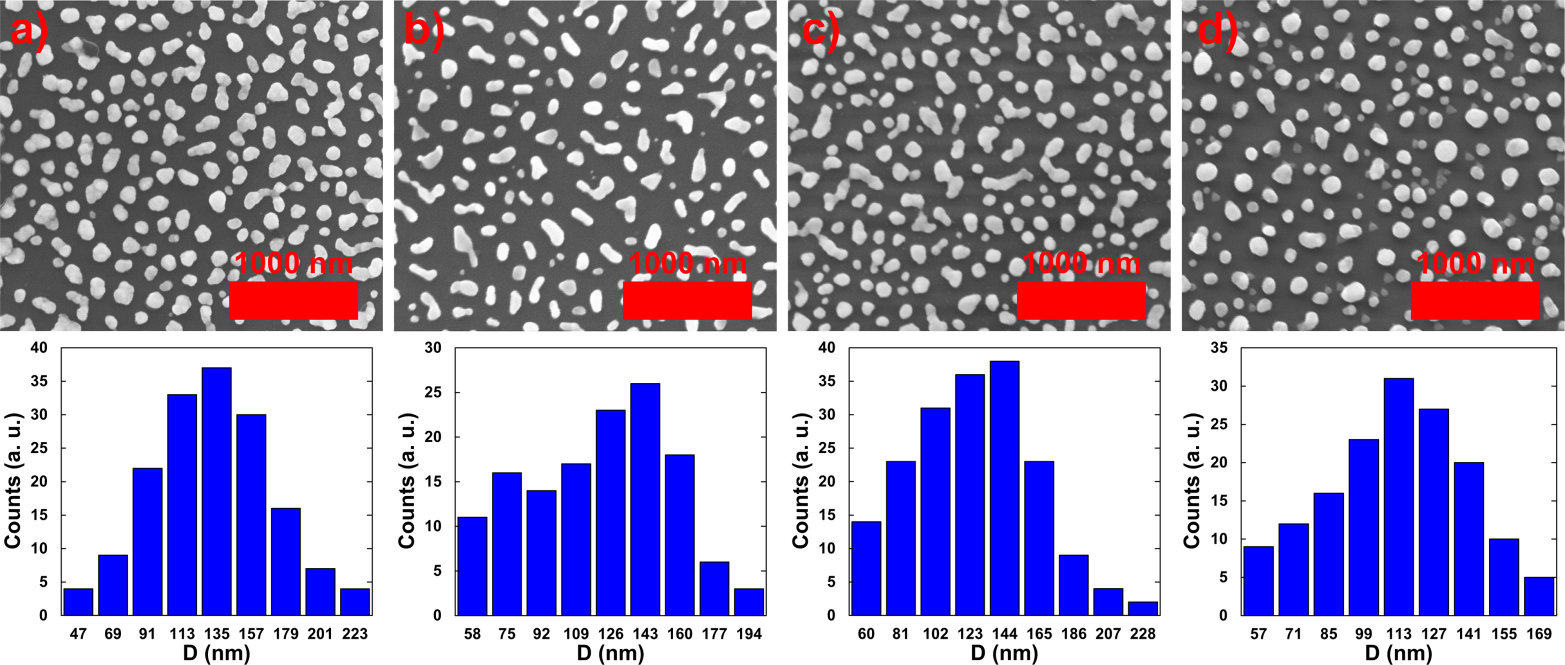
SEM pictures of nanostructures and calculated island diameter distribution resulting from annealing: a) 4 nm Ag and 4 nm Pt bilayer at 650 °C for 15 min, and 5 nm Ag and 5 nm Pt bilayer for 15 min. at: b) 650 °C, c) 675 °C, d) 700 °C.

To further investigate the thermal decomposition behavior, Ag/Pt bilayers with increased individual layer thicknesses of 5 nm each were also examined. This approach was expected to provide a wider range and greater control over the final alloy composition. However, SEM observations revealed that an annealing temperature of 650 °C was insufficient to produce spherical, isolated nanostructures, instead resulting in the formation of irregularly shaped nanoislands. Therefore, the annealing temperature was gradually increased. At 700 °C, well-developed, isolated metallic islands with nearly spherical shapes were obtained, indicating a more advanced stage of morphological transformation. SEM images of the nanostructures formed after annealing at 650 °C, 675 °C, and 700 °C are presented in Figure 3a–c. In Figure 3c, in addition to the clearly visible nanostructures, lighter gray, triangular-shaped islands can also be observed on the surface. These features may originate from the formation of metallic or metal–silicon compound flakes during annealing at higher temperatures. It may also be associated with partial sublimation or evaporation of the material at 700 °C. Similar triangular flakes have been previously reported during the formation of, for example, Ag [48], MoS_2_ [49], or WS_2_ [50]. Additionally, the chemical composition of the nanostructures manufactured at 700 °C was measured by EDS. The spectrum presented in Figure 3d confirms the presence of Ag and Pt. The Ag and Pt ratio was calculated to be approximately 40 atom %:60 atom %. This reduced fraction may result from partial desorption of silver. Sublimation of Ag atoms plays a significant role in the morphology and the evolution of the elemental composition of the nanostructures. Due to the higher vapor pressure of Ag atoms, they can preferentially desorb from the nanostructures, and the rate of Ag sublimation exponentially increases with temperature [51–53]. Additionally, based on lower-magnification SEM images of the nanostructures, we determined the size distribution of the formed structures, which is presented in Figure 4. It can be observed that annealing the 4 nm Ag/4 nm Pt bilayer at 650 °C for 15 min results in the relatively small average nanostructure size and the well uniform size distribution. This observation prompted the selection of a series with a total bilayer thickness of 8 nm, fabricated at 650 °C, for further investigations.

**Figure 5:**
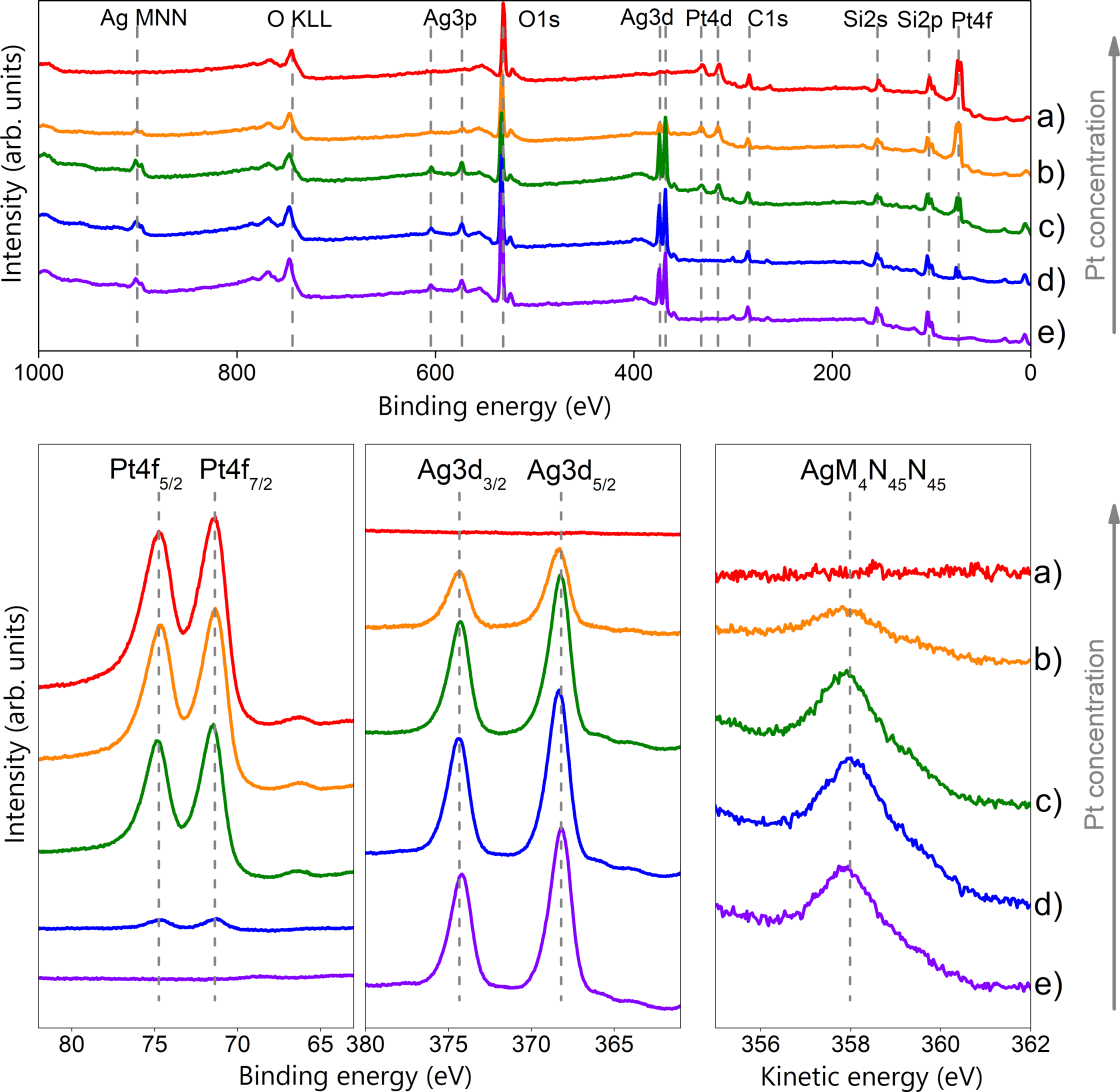
XPS survey and the high resolution spectra of Pt 4f, Ag 3d and Ag, and 3d Ag Auger line regions of the nanostructures obtained by annealing for 15 min at 650 °C metallic bilayers with initial thickness of: a) 8 nm Pt, b) 4 nm Ag/4 nm Pt, c) 6 nm Ag/2 nm Pt, d) 7 nm Ag/1 nm Pt, e) 8 nm Ag.

A detailed chemical composition analysis of pure Ag and Pt nanostructures, as well as those synthesized with various Ag–Pt ratios, was performed using X-ray photoelectron spectroscopy (XPS) and is shown in Figure 5. The survey spectra confirm the high chemical purity of all samples. Apart from Ag and Pt, only silicon originating from the substrate, as well as oxygen and carbon adsorbed from the atmosphere, were detected. The quantitative composition was determined from the survey spectra and is presented below in Figure 10b. The obtained values showed a good agreement with the theoretical compositions, in line with the EDS measurements. To determine a detailed chemical state analysis of the elements, high-resolution spectra were also recorded. The position of Pt 4f_7/2_ peaks at 71.2 eV and a characteristic doublet with a splitting energy equal to 3.3 eV is consistent with metallic Pt [54–55]. The Ag 3d_5/2_ and Ag 3d_3/2_ photoelectron peaks were detected at ca. 368.2 eV and 374.2 eV, respectively, and the spin–orbit splitting was equal to 6 eV, which is characteristic of silver. However, distinguishing between different silver chemical states solely from the position of the 3d_5/2_ peak is difficult. For this reason, the Auger parameter was calculated according to the formula: α = BE + KE, where BE is the binding energy of the Ag3d_5/2_ peak and KE is the kinetic energy of the Ag M_4_N_45_N_45_ peak [56–57]. To calculate the parameter, the kinetic energies of the Ag M_4_N_45_N_45_ Auger lines were recorded (Figure 5). Their positions were detected at ca. 357.8 eV for all samples. The calculated parameter is equal to 726 ± 0.1 eV, which perfectly matches the reference value for metallic silver [57–59].

**Figure 6:**
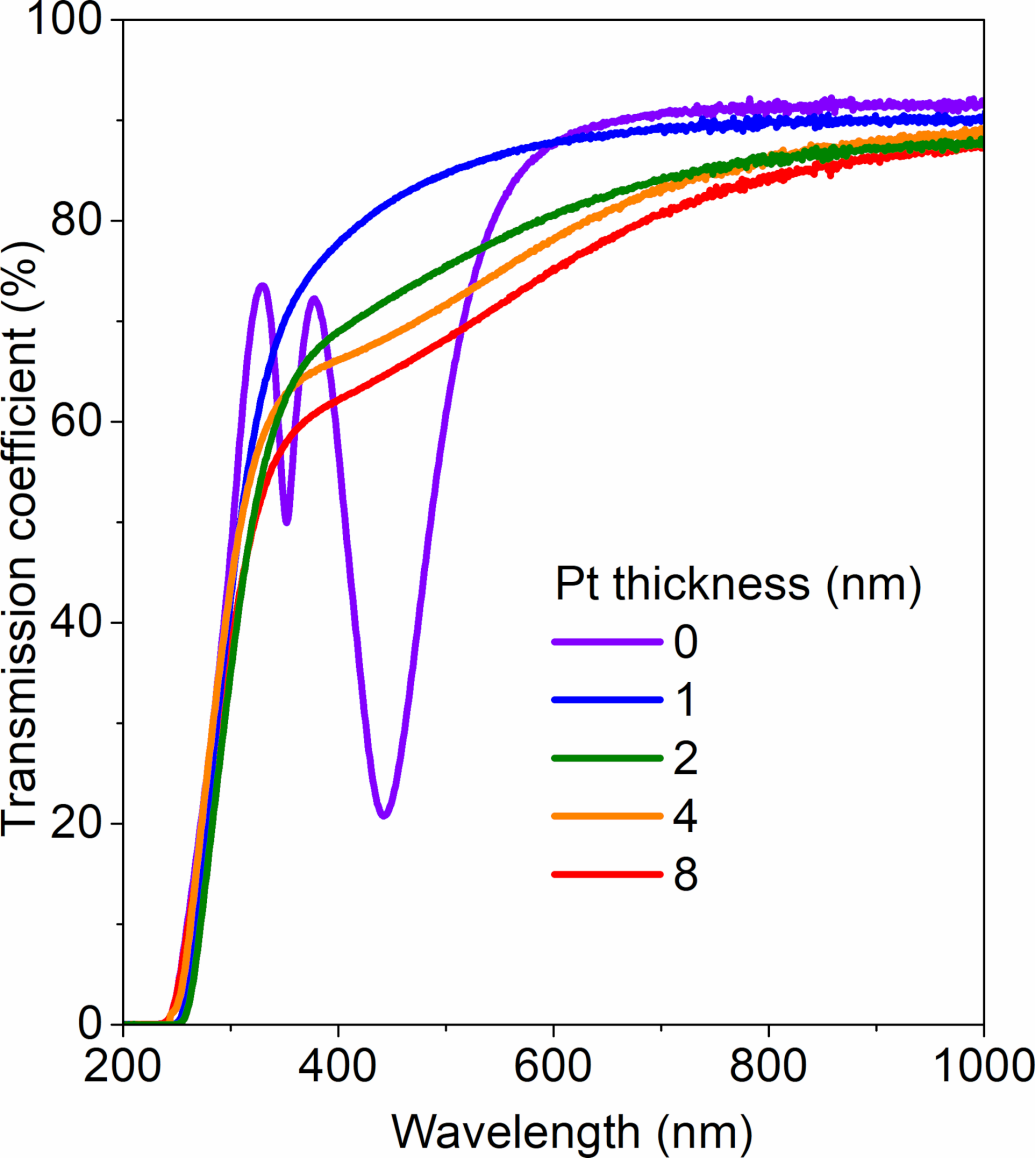
Transmittance spectra of Ag–Pt nanostructures formed by annealing at 650 °C for 15 min, bilayers with a total thickness of 8 nm and varying relative thicknesses of the Ag and Pt films.

**Figure 7:**
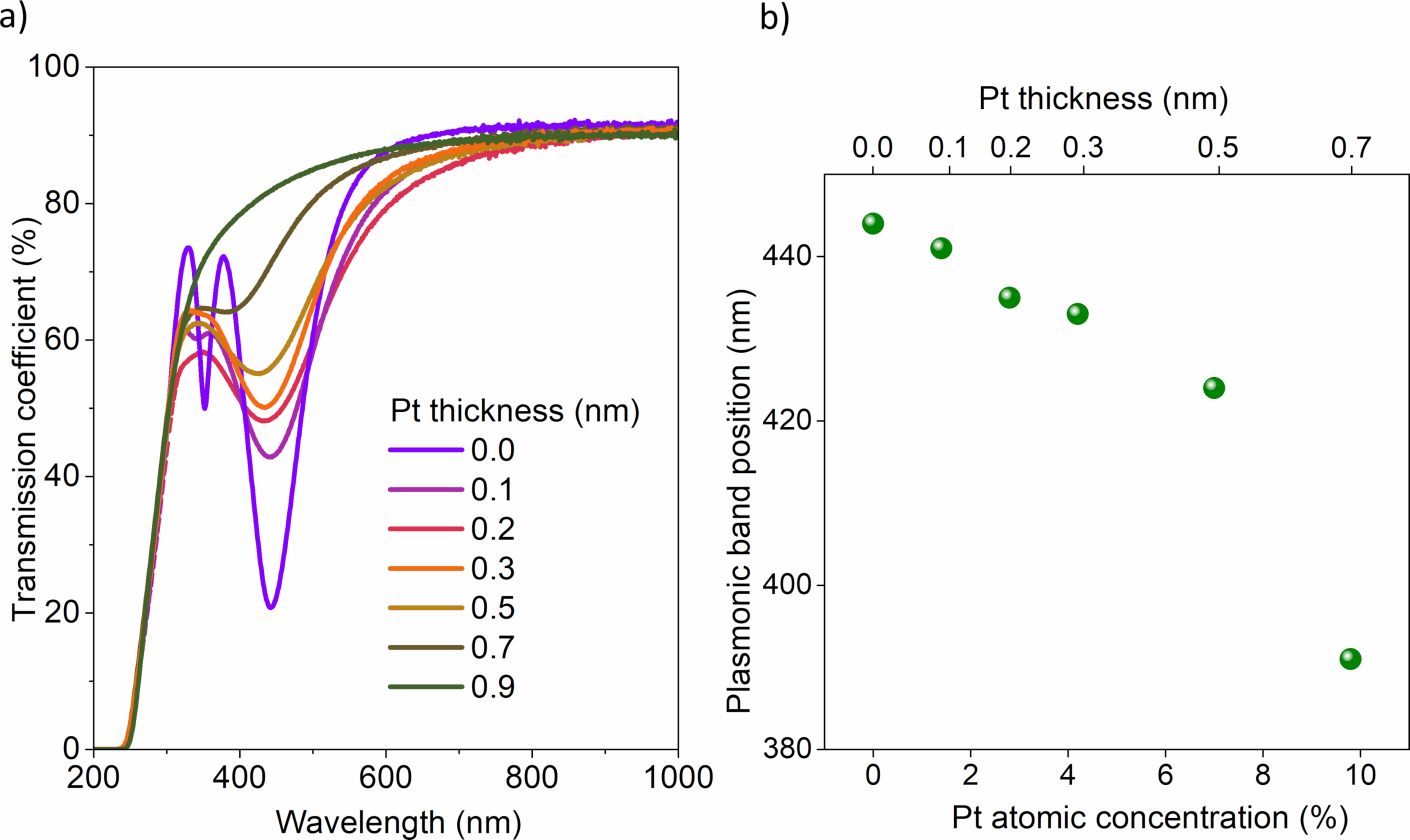
a) Transmittance spectra of Ag–Pt nanostructures formed by annealing at 650 °C for 15 min, bilayers with total thickness of 8 nm and varying relative thicknesses of the Ag and Pt films. b) Position of the plasmonic band of Ag–Pt nanoalloy structures as a function of composition. Each measured structure was fabricated either from a single Ag layer or bilayers with a Pt film deposited on top of an Ag layer. The total thickness of such systems was 8 nm. All samples were subsequently annealed at 650 °C for 15 min.

The formation of nanoalloys was verified using UV–vis spectroscopy. This technique is rapid and straightforward, yet sufficiently sensitive to confirm whether alloying at the nanoscale has occurred. In nanomaterials, distinguishing between a nanoalloy and a nanocomposite is often not trivial, as the boundary between these two classes is not always clearly defined. Examination properties, such as optical properties, can provide a valuable criterion for differentiation. In plasmonic materials, in most cases, nanocomposites exhibit two distinct resonance peaks, each corresponding to one of the constituent phases. In contrast, nanoalloys typically show a single resonance peak, indicating the formation of a homogenously mixed metallic phase at the nanoscale [26,60]. Our previous investigations confirmed these differences. Within the Ag–Au nanostructures, nanoalloy formation was clearly observed. By systematically adjusting the elemental composition, we achieved controlled modulation of the plasmonic band position [25]. On the other hand, in the Ag–Cu system, the material behaved as a nanocomposite, with two clearly distinguishable resonance peaks attributable to the individual components [26]. From Figure 6, it can be seen that the transmission spectra do not exhibit a plasmonic band. Only in the spectrum measured for pure silver nanostructures, a well-defined surface plasmon resonance band is clearly visible, accompanied by an additional minimum attributed to the quadrupole resonance [26,61]. Increasing the platinum content results in a slight decrease in the overall transmission; however, no plasmonic resonance is observed. On the other hand, when examining very small variations in composition, within a range of only a few atomic percent of Pt, the plasmonic resonance of Ag–Pt alloyed nanostructures can be observable (Figure 7a). Above approximately 10 atom % Pt, the resonance completely disappears. A pronounced blue shift of the resonance band, particularly visible in Figure 7b, accompanied by a rapid smoothing of the quadrupolar resonance peak, is clearly visible for pure silver and for nanostructures formed by the disintegration of a 7.9 nm Ag/0.1 nm Pt multilayer. The blue shift is expected upon alloying silver with platinum, since the plasmon resonance for Pt occurs in the near-UV region [62–65]. The rapid suppression of the plasmonic response with increasing Pt concentration arises from the intrinsic electronic properties of platinum, whose Fermi level lies close to partially filled d-states. These d-electron transitions strongly enhance interband absorption, leading to damping of interband sp excitations and broadening of the resonance band. In consequence, it causes an increase in both the real and imaginary components of the dielectric function (ε_1_ and ε_2_), leading to the quenching of the plasmon resonance [66]. Moreover, Pt d-electrons do not participate in the collective charge density oscillations in Ag–Pt alloys [37,67–68].

**Figure 8:**
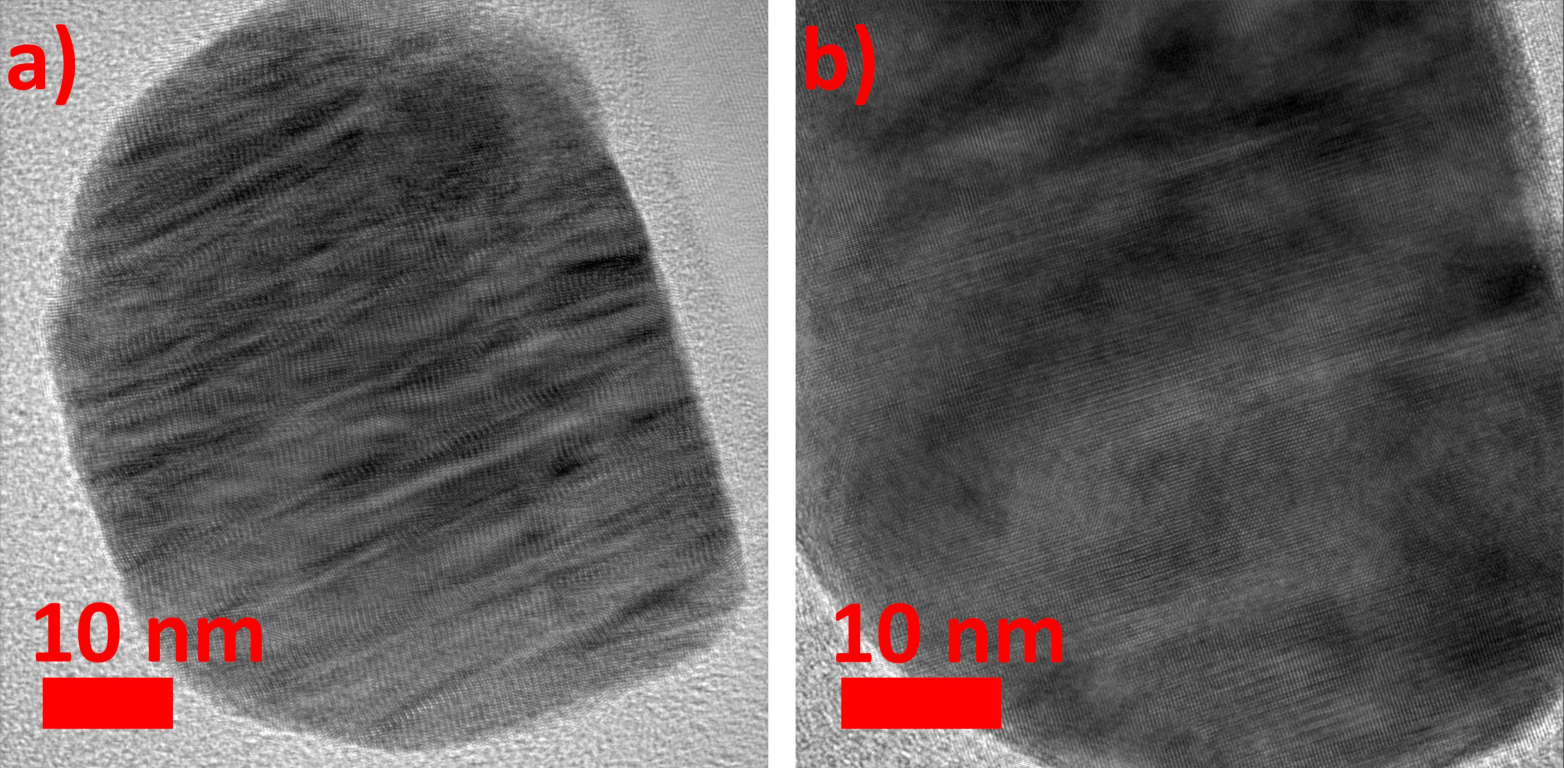
HR-TEM image of a cross-section of a nanoisland obtained by annealing at 650 °C for 15 min, bilayers with a thickness of: a) 4 nm Ag/4 nm Pt and b) 7 nm Ag/1 nm Pt.

**Figure 9:**
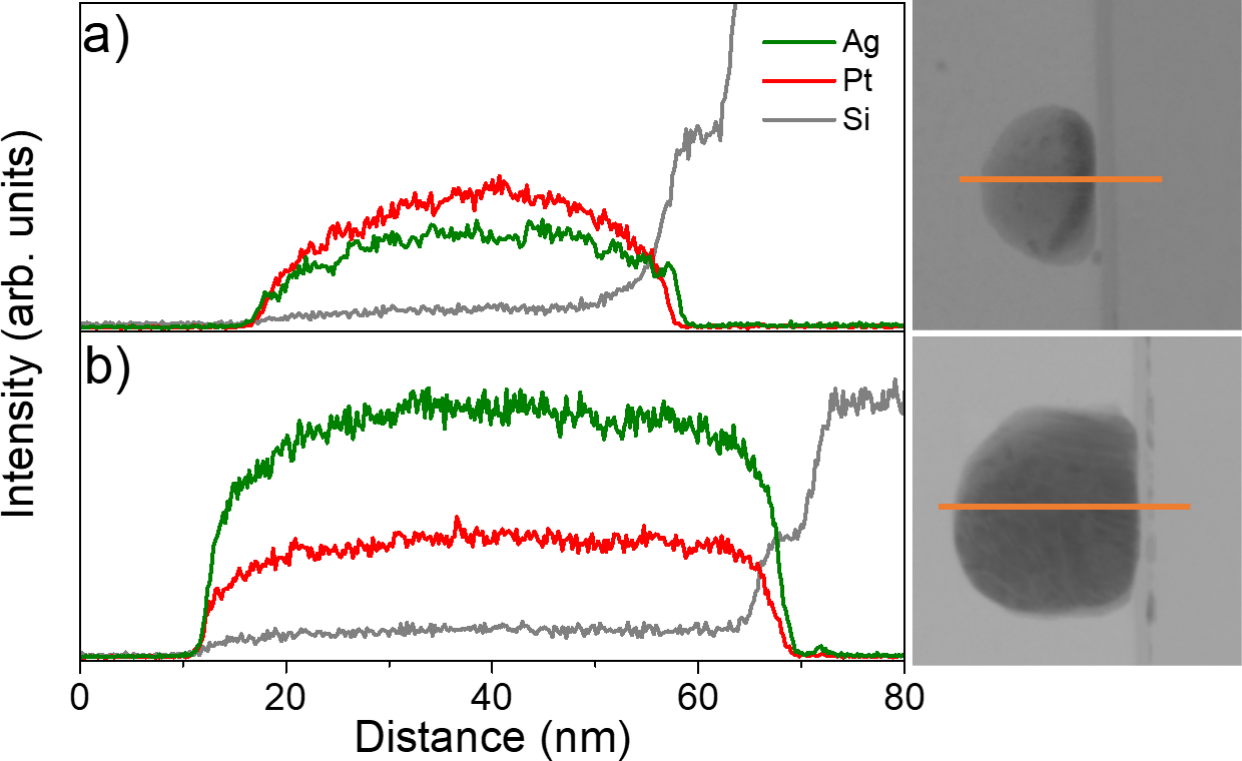
Detailed EDS analysis of the cross-section of the nanoisland obtained by annealing at 650 °C for 15 min, bilayers with a thickness of: a) 4 nm Ag/4 nm Pt and b) 7 nm Ag/1 nm Pt.

**Figure 10:**
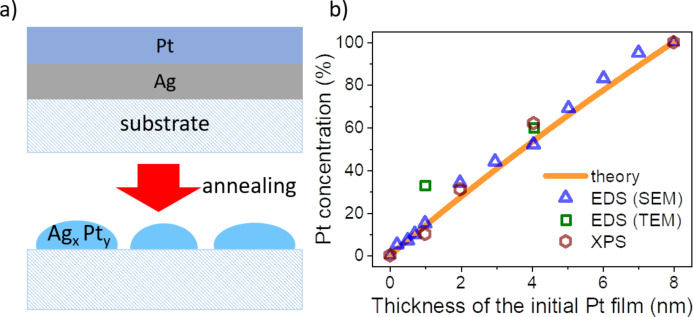
a) Schematic illustration of the fabrication process of alloy nanostructures. b) Comparison of the theoretical and measured composition of nanostructures; EDS (SEM) measured by EDS integrated with an SEM microscope, EDS (TEM) measured by an analyzer integrated with TEM, and by XPS.

To further confirm the formation of homogeneous Ag–Pt nanoalloys, high-resolution transmission electron microscopy (HR-TEM) observations were performed. Representative cross-section TEM images of two samples with various compositions are shown in Figure 8. Images for both nanostructures exhibit uniform morphology, which strongly indicates alloy formation rather than phase-separated Ag and Pt regions. A detailed EDS analysis was performed on the cross-sections using both line scans and area modes. For the structure fabricated from 4 nm Ag and 4 nm Pt, the measured composition reached approximately 60 atom % Pt and 40 atom % Ag. The sample prepared from 7 nm Ag and 1 nm Pt exhibited a composition of about 33 atom % Pt and 67 atom % Ag. In both cases, the platinum content was noticeably higher than the nominal ratios expected from the initial layer thicknesses. Measured data were presented and compared with other results below in Figure 10b. These results align with previous findings and further confirm significant Ag desorption, even at the annealing temperature of 650 °C. The linear EDS scans presented in Figure 9 confirm the complete homogeneity of the formed nanostructures, showing no significant dominance of either element at any specific location.

Regarding the phase equilibrium system of Ag–Pt, the scientific literature reveals considerable discrepancies, which have been analyzed in detail in [28]. In general, agreement exists only with respect to the liquidus, the peritectic reaction, and the presence of a wide miscibility gap in the solid phase. However, based on our HR-TEM investigations (Figure 8a), it can be concluded that our results qualitatively confirm the presence of an ordered phase in the vicinity of the Ag–Pt composition (4 nm Ag/4 nm Pt). The modulation observed in the HR-TEM images may be attributed to either regions of differing crystallographic orientations or to the presence of phases with varying stoichiometry (degree of ordering). In contrast, nanostructures with Ag-rich compositions (7 nm Ag/1 nm Pt), as shown in the HR-TEM images (Figure 8b), exhibit a regular crystalline ordering of Ag_1−_*_x_*Pt*_x_* alloys, modifying the base crystal structure of Ag.

It should be emphasized, however, that the investigated nanostructures were formed within the compositional range corresponding to a solid-phase miscibility gap at a temperature of 600 °C. Moreover, they were dynamically generated during the dewetting process from Ag and Pt components characterized by significantly different melting temperatures and surface energies (961 °C and 1.25 J/m^2^ for Ag; 1768 °C and 2.48 J/m^2^ for Pt). Additionally, pronounced Ag segregation occurring during nanostructure formation may strongly influence the degree of chemical ordering in Ag–Pt nanoalloys, as discussed in [69–70].

The linear EDS analysis also confirmed the formation of a thin intermediate layer, resulting from the diffusion of silicon from substrate into the structures. A similar effect was previously observed in Ag–Au nanostructures, where we noticed diffusion through the native silicon oxide film, as reported in [25], as well as in studies on ultrathin Al_2_O_3_ films described in [71].

## Conclusion

As shown by the presented results, the applied fabrication method enables the formation of a nanoalloy composed of elements that do not form alloys on the micro- or macroscale. This is particularly important when considering potential applications of such nanoalloys, for instance, in catalysis or nanosensing. The Ag–Pt system is particularly noteworthy because of its high gas absorption capacity, making it highly relevant for catalytic applications. In this study, a method for manufacturing Ag–Pt nanoalloys by thermally activated disintegration, also known as dewetting of thin metallic films, was successfully demonstrated. The dewetting approach is auspicious due to its simplicity and cost-effectiveness. This process exploits the inherent instability of continuous metallic nanoscale films, resulting in their gradual breakup and the formation of isolated nanoislands. Furthermore, the ability to obtain homogeneous Ag–Pt nanoalloys at the nanoscale, despite their immiscibility in the bulk form, demonstrates that the proposed method effectively overcomes the limitations of conventional alloying techniques and opens new pathways for designing functional nanomaterials.

Annealing the Pt on Ag bilayers (with a total thickness of 8 nm) at a temperature of 650 °C for 15 min resulted in the formation of well-defined, nearly spherical, isolated nanoislands. Chemical analysis using EDS spectroscopy confirmed the coexistence of Ag and Pt within the nanostructures. However, a noticeable deficiency of Ag was observed, which can be attributed to silver desorption. The chemical state of the elements, as determined by XPS, confirmed their metallic character, and the obtained concentration values were in good agreement with the EDS measurements. Detailed HR-TEM microscopy investigations showed that the Ag–Pt nanoalloys are perfectly homogeneous. The cross-sectional images of the nanostructures exhibited a uniform structure, which strongly suggests the formation of an alloy. These observations were supported by linear EDS scans on the cross-sections, which confirmed the complete homogeneity of the formed nanostructures. Verification of Ag–Pt nanoalloy formation was also confirmed using the UV–vis spectroscopy method by observing the extinction of the Ag plasmon resonance by the addition of Pt.

## Experimental

Corning 1737 glass and Si (111) substrates covered by a native oxide layer were used for the deposition of platinum/silver bilayers. Samples on Si substrates were used for detailed characterization of surface morphology and chemical composition, whereas Corning glass substrates were employed for optical investigations. Both types of substrates were cleaned with acetylacetone, subsequently rinsed with ethanol, and dried. Thin Pt and Ag thin films were sputtered using a table-top DC magnetron sputtering coater (EM SCD 500, Leica) in a pure Ar plasma environment (Argon, Air Products 99.99%). Both Pt and Ag targets used in the deposition process were 99.99% pure. The coating procedure was performed at a deposition rate of approximately 0.10 nm/s and 0.07 nm/s for Ag and Pt films, respectively. The applied power was in the range of 30–35 W. A quartz crystal microbalance integrated within the sputtering system allowed for in situ monitoring of film thickness. Ag–Pt nanoalloyed structures were prepared by sequential deposition of Ag and Pt thin films, as shown in Figure 10a. In this process, Pt was deposited onto Ag due to the lower chemical reactivity of platinum, which additionally provides protection against oxidation of deposited bilayers. The composition of the nanostructures was adjusted by varying the thickness of the initial metallic films, according to the dependence shown in Figure 10b. This relationship was established based on theoretical stoichiometric calculations taking into account the film thicknesses, densities, molar masses, and resulting atomic concentrations of the constituent metals. The total thickness of the deposited multilayer was maintained at 8 nm, which constituted the main measurement series. Due to the well-known plasmonic properties of pure silver structures and the significantly stronger extinction cross section (which can be considered a measure of the resonance response strength to excitation by an external electromagnetic field), observed for Ag compared to Pt, the number of samples was increased in the range of very low platinum content [61–62,72]. In addition, the suppression and a near UV shift of the resonance in the case of Pt further justified this approach [62–64]. The deposited layers were subjected to thermal annealing in a hot furnace under an argon atmosphere (Argon, Air Products, 99.99%) for 15 min at a temperature of 650 °C to induce the formation of nanostructures. Both deposition and annealing parameters were selected based on our prior research on metallic nanostructures [23–26,51,61,73].

The microstructure of as-deposited Ag–Pt bilayers was determined by means of AFM with the use of NT-MDT Ntegra AFM. To obtain high-resolution, ultra-sharp probes with a tip radius <5 nm were used. Measurements were conducted in the semi-contact mode. The morphology of the manufactured nanostructures was examined using microscopic techniques. A FEI Quanta FEG 250 scanning electron microscope operating at 10 kV was used to observe the formation of nanostructures and to analyse the surface morphology of the samples. Moreover, an energy dispersive X-ray spectrometer was employed for elemental analysis of the isolated islands, providing a fast and preliminary insight into the nanostructure formation. The chemical composition of the nanostructures was confirmed using XPS. Measurements were carried out with a hemispherical XPS spectrometer (Argus, Omicron Nanotechnology) equipped with an Mg Kα X-ray source, operated at 15 keV and 300 W. XPS measurements were performed under ultra-high vacuum at room temperature, with a pressure below 1.1 × 10^−8^ mbar. The results were calibrated to the C 1s line and analyzed using Shirley background subtraction and a Gaussian–Lorentzian curve as the fitting algorithm by the CasaXPS software. Additionally, detailed investigations of the microstructure and chemical composition of the single nanostructure cross-sections were conducted using TEM. The studies were performed using a JEOL JEM-F200 TEM operated at 200 keV and equipped with a dual SDD EDS system, which facilitates the observation of nanoalloy formation. Thin foils for TEM observations were prepared using the focused ion beam (FIB) lift-out technique. For this purpose, a dual-beam Thermo Fisher Scientific Helios 5 UX scanning electron microscope equipped with a Phoenix Ga^+^ ion column was used. Prior to sample preparation, the particles were coated with a protective carbon layer using a gas injection system (GIS). The final thinning of the samples was conducted by gradually reducing the acceleration voltage to 2 kV, thereby minimizing Ga-induced defects. The formation of the nanoalloys was confirmed by measuring their optical properties, with particular emphasis on plasmonic behavior. These properties were studied using UV–vis spectroscopy with a Thermo Fisher Scientific Evolution 220 double-beam spectrophotometer in transmittance mode over the range of 200–1000 nm.

## Data Availability

Data generated and analyzed during this study is openly available in mostwiedzy.pl at https://doi.org/10.34808/6bvs-y478.
